# Differential neural responses to naturally occurring envelopes in the electrosensory system

**DOI:** 10.1186/1471-2202-15-S1-P192

**Published:** 2014-07-21

**Authors:** Chengjie Huang, Maurice J Chacron

**Affiliations:** 1 Department of Physiology, McGill University, Montreal, Quebec H3G 1Y6, Canada; 2 Department of Physics, McGill University, Montreal, Quebec H3G 1Y6, Canada

## 

Natural sensory stimuli frequently consist of a fine structure whose amplitude (i.e. envelope) varies more slowly. Previous studies have demonstrated that envelope signals are found across sensory systems and are necessary for perception. For example, envelope signals in the auditory system carry information pertaining to the perception of pitch fluctuations in communication vocalizations [1], while envelopes in the visual system are responsible for object grouping and the perception of illusory contours [2,3]. However, the neural mechanisms underlying the encoding of these envelope signals are poorly understood in general. Gymnotiform wave-type weakly electric fish constitute an attractive model system for studying envelope processing in the brain because of its well-characterized anatomy and physiology [4]. These fish generate around themselves a weak quasi-sinusoidal electric field through the electric organ discharge (EOD) [5]. Recent studies have shown that the beat amplitude (i.e. the envelope) can vary in time during different behavioral contexts and display differential frequency content: while envelopes caused by movement primarily contain low (<1 Hz) temporal frequencies [6], those caused by social interaction instead contain higher (>1 Hz) temporal frequencies [7]. While it is known that electrosensory neurons can respond to envelopes [7], whether these neurons respond differentially to envelopes arising from movement and social interaction is unknown in part because their tuning to envelope frequencies has not been characterized. Here we used well established techniques [8] to record from electrosensory pyramidal neurons across different maps of the body surface within the electrosensory lateral line lobe (ELL) in response to envelope stimuli with different frequency content spanning the behaviorally relevant range (0.05-10 Hz). Using standard measures of linear systems identification techniques as well as more quantitative methods of information theory, we found that pyramidal neurons in different maps displayed differential responses to envelopes arising from different behavioral contexts. Our results suggest that information about envelopes arising from different behavioral contexts begins to be segregated at the level of the ELL and provide new insights as to how these behaviorally relevant stimuli are encoded in early sensory pathways.

## References

[B1] Burns EM Viemeister NF Played-again SAM: Further observations on the pitch of amplitude-modulated noise Journal of Acoustical Society of America 1981 15 1655 1660 10.1121/1.387220

[B2] Shapley R Visual cortex: pushing the envelope Nature neuroscience 1998 15 2 95 96 10.1038/342 10195120

[B3] Grosof DH Shapley RM Hawken MJ Macaque V1 neurons can signal 'illusory' contours Nature 1993 15 6446 550 552 10.1038/365550a0 8413610

[B4] Maler L Receptive field organization across multiple electrosensory maps. I. Columnar organization and estimation of receptive field size The Journal of comparative neurology 2009 15 5 376 393 10.1002/cne.22124 19655387

[B5] Bennett MV Electrolocation in fish Annals of the New York Academy of Sciences 1971 15 242 269 10.1111/j.1749-6632.1971.tb13102.x 4331616

[B6] Fotowat H Harrison RR Krahe R Statistics of the electrosensory input in the freely swimming weakly electric fish Apteronotus leptorhynchus The Journal of neuroscience : the official journal of the Society for Neuroscience 2013 15 34 13758 13772 10.1523/JNEUROSCI.0998-13.2013 23966697 PMC6618647

[B7] Stamper SA Fortune ES Chacron MJ Perception and coding of envelopes in weakly electric fishes The Journal of experimental biology 2013 15 Pt 13 2393 2402 23761464 10.1242/jeb.082321 PMC4529321

[B8] Deemyad T Metzen MG Pan Y Chacron MJ Serotonin selectively enhances perception and sensory neural responses to stimuli generated by same-sex conspecifics Proceedings of the National Academy of Sciences of the United States of America 2013 15 48 19609 19614 10.1073/pnas.1314008110 24218585 PMC3845146

